# Gene expression comparison reveals distinct basal expression of HOX members and differential TNF-induced response between brain- and spinal cord-derived microvascular endothelial cells

**DOI:** 10.1186/s12974-016-0749-6

**Published:** 2016-11-10

**Authors:** Yves Molino, Françoise Jabès, Amandine Bonnet, Nicolas Gaudin, Anne Bernard, Philippe Benech, Michel Khrestchatisky

**Affiliations:** 1 Vect-Horus SAS, Faculté de Médecine - Secteur Nord, 51 Bd Pierre Dramard, 13344 Marseille Cedex 15, France; 2 Aix Marseille Univ, CNRS, NICN, Marseille, France

**Keywords:** Microvessels, Endothelial cells, Vascular beds, HOX members, TNF-α response, Matrix metalloproteinase (MMP)

## Abstract

**Background:**

The heterogeneity of endothelial cell types underlies their remarkable ability to sub-specialize and provide specific requirements for a given vascular bed. Here, we compared rat microvascular endothelial cells (MECs) derived from the brain and spinal cord in both basal and inflammatory conditions.

**Methods:**

We used whole rat genome microarrays to compare, at different time points, basal and TNF-α-induced gene expression of rat MECs from in vitro models of the blood-brain barrier (BBB) and blood-spinal cord barrier (BSCB). Validation at both messenger RNA (mRNA) and protein levels was performed on freshly extracted microvessels (MVs) from the brain and spinal cord (BMVs and SCMVs, respectively), as these were considered the closest in vivo tissues to cultured MECs.

**Results:**

Most of the genes encoding adhesion/tight junction molecules and known endothelial markers were similarly expressed in brain and spinal cord MECs (BMECs and SCMECs, respectively). However, one striking finding was the higher expression of several *Hox* genes, which encode transcription factors involved in positional identity. The differential expression of *Hoxa9* and *Hoxb7* at the mRNA levels as well as protein levels was confirmed in BMVs and SCMVs. Although the TNF-α response was in general higher in BMECs than in SCMECs at 12 h, the opposite was observed at 48 h. Furthermore, we found that expression of *Tnfrsf1a and Tnfrsf1b* encoding the TNF receptor super-family member 1a/TNFR1 and 1b/TNFR2, respectively, were constitutively higher in BMVs compared to SCMVs. However, only *Tnfrsf1b* was induced in SCMECs in response to TNF-α at 24 and 48 h.

**Conclusions:**

Our results support a role for HOX members in defining the positional identities of MECs in vivo. Our data also suggest that the delayed transcriptional activation upon TNF-α treatment in SCMECs results from the requirement of the TNF-induced expression of *Tnfrsf1b*. In contrast, its high basal expression in BMECs might be sufficient to confer an immediate and efficient TNF-α response.

**Electronic supplementary material:**

The online version of this article (doi:10.1186/s12974-016-0749-6) contains supplementary material, which is available to authorized users.

## Background

The brain and spinal cord are endowed with particular vascular systems, known as the blood-brain barrier (BBB) and blood-spinal cord barrier (BSCB), respectively, which maintain homeostasis between nervous parenchyma and peripheral circulation. These barriers are composed of microvascular endothelial cells (MECs) and neighboring elements of the neuro-glia-vascular unit (NGVU) such as pericytes, astrocytic end-feet processes, and neurons. The two vascular systems share physical and physiological barrier properties including basement membranes, highly differentiated tight junctions (TJs), low levels of endocytosis and vesicular transport, a broad spectrum of molecular pumps, polarized carriers, and receptors involved in transcytosis mechanisms [1, 2].

Despite these common features, the BSCB presents structural and functional differences resulting in distinct vulnerability to pathological insults when compared to the BBB [2, 3]. For instance, the microvessels (MVs) of the spinal cord contain glycogen deposits, which are not normally seen in the cerebral MVs [4]. Only limited populations of brain astrocytes express the phosphorylated form of glial fibrillary acidic protein (GFAP) while almost all astrocytes in the spinal cord do so [5]. Most in vivo studies describe uniform presence of pericytes in various brain regions, while the spinal cord presents with 70 % less pericytes compared to the brain, with a non-uniform distribution along the rostrocaudal extent of the spinal cord, the thoracic region being richer in pericytes [6, 7]. In vitro, brain and spinal cord pericytes differ markedly in their potential for tube formation and migration, reflecting more differences between the BBB and BSCB [8]. Such differences in their microenvironment may in turn induce MEC heterogeneity between brain and spinal domains, correlating with a higher BSCB inherent permeability [9, 10]. This increased permeability might result from differences in cell junction protein expression between BBB and BSCB endothelial cells. In cultured SCMECs compared to BMECs, TJ proteins ZO-1 and occludin expression levels are decreased, while claudin-1 and claudin-5 remain unchanged, confirming that this decrease is specific [11]. Adherens junction proteins such as VE-cadherin and β-catenin also show reduced expression in SCMVs and cultured SCMECs [11].

Like their brain counterparts, SCMECs are involved in pathological processes associated with many neurological conditions [3]. Although, the structural and functional differences might explain that certain disease states differentially affect BMEC and SCMEC populations, very little is known concerning their intrinsic differences, including their response to inflammation. To gain more insight into such differences, a comparative transcriptomic analysis was performed on RNAs extracted from MEC monolayers of in vitro models of the BBB and BSCB [12] left untreated or treated with TNF-α for 12, 24, and 48 h.

## Methods

### Animals

Procedures involving animals conform to National and European regulations (EU directive No. 2010/63) and to authorizations delivered to our animal facility (No. C13 055 08) and to the project (No. 00757.02) by the Local Ethics Committee and French Ministry of Research. All efforts were made to minimize animal suffering and reduce the number of animals used. Wistar rats were obtained from Elevage Janvier (St Berthevin, France).

### Rat syngeneic in vitro BBB and BSCB models

The production of in vitro BBB and BSCB models is based on our previously described protocol [12]. Briefly, primary cultures of BMECs and SCMECs, prepared from 5- to 6-week-old Wistar rats, were seeded in the luminal compartment of six-well plate polyethylene insert filters (Merck Millipore, Billerica, MA, USA), pre-coated with collagen type IV and fibronectin (BD Biosciences, Franklin Lakes, NJ, USA) to establish the endothelial cell monolayers. Astrocytes, prepared from neonatal Wistar rats, were seeded in the bottom of the six-well plates and co-cultured with the endothelial cell monolayers in endothelial cell media (ECM) containing DMEM/F12 supplemented with 20 % bovine platelet poor plasma derived serum (Alfa Aesar, Ward Hill, MA, USA), basic fibroblast growth factor (bFGF) 2 ng/mL, heparin 100 μg/mL, gentamycin 50 μg/mL, HEPES 2.5 mM, and hydrocortisone 500 nM (all from Life Technologies, Carlsbad, CA, USA). Under these conditions, the BMEC and SCMEC monolayers differentiate, express junction-related proteins within 3 days, and remain optimally differentiated during three more days.

### Induction and assessment of inflammation

#### Cytokine production

The day of the experiment, the inserts containing the BMEC and SCMEC monolayers were transferred to new six-well plates without astrocytes and stimulated for 12, 24, and 48 h with recombinant rat TNF-α 5 ng/mL (Peprotech, Rocky Hill, NJ, USA). Supernatants were collected, centrifuged, and stored at −80 °C until analysis. Rat CCL2 levels were evaluated using commercially available ELISA kits (Peprotech) according to the manufacturer’s instructions. All samples were analyzed in duplicates.

#### Transport assay

After 24 h inflammation with recombinant rat TNF-α (5 ng/mL, Peprotech), barrier integrity of the in vitro models was controlled with Lucifer Yellow (LY CH lithium salt, Sigma Aldrich), a small hydrophilic molecule (MW 457 g/mol) retained by the monolayers as previously described [12]. Briefly, quantification of the LY paracellular leakage from the luminal to the abluminal compartment was assessed by fluorimetric analysis (excitation at 430 nm and emission at 535 nm) and expressed in LY permeability, Pe_(LY)_. Barrier integrity was validated for Pe_(LY)_ below 0.6.10^−3^ cm/min.

#### Immunocytochemistry

The MEC monolayers were washed three times with PBS 1× (Life Technologies) and gently dissociated from the insert filters followed by a 20-min fixation in paraformaldehyde (PFA) 4 % (*w*/*v*) (Sigma Aldrich, Saint-Louis, MO, USA) prior to immunocytochemistry. After three washes with PBS 1× (Life Technologies), the cells were pre-incubated for 30 min at room temperature (RT) with blocking buffer containing BSA 3 % (PAA Laboratories, Velizy-Villacoublay, France) in PBS 1×. The MEC monolayers were stained for 1 h in PBS 1× containing BSA 1 % (PAA Laboratories), with saponine 0.1 % (Sigma Aldrich) for membrane permeabilization, with a rabbit anti-occludin 1.5 μg/mL (Life Technologies). Cell nuclei were labeled with Hoechst 33342 1/1000 (Life Technologies) in co-incubation with a donkey anti-rabbit Alexa Fluor 488 secondary antibody (Jackson Immunoresearch, West Grove, PA, USA). Cells were washed and mounted in Prolong Gold antifade mounting medium (Life Technologies). The mounted slides were observed with a Leica TCS SP2 confocal microscope (Leica Microsystems, Heidelberg, Germany). High-magnification images were acquired using a 63X HCX PL APO oil immersion objective and analyzed using the NIH ImageJ software (version 1.49o for Mac).

### Tissue sampling

The BMEC and SCMEC monolayers (stimulated or not with TNF-α) were pre-incubated on ice with a solution of DPBS 1× (without calcium and magnesium) and EDTA 0.25 mM (both from Life Technologies), then mechanically detached with a cell scraper and centrifuged at 1200×*g* for 10 min. The BMVs and SCMVs were prepared from 5- to 6-week-old Wistar rats according to our previously described protocol [12]. Instead of plating, they were washed with DPBS 1× (Life Technologies) and centrifuged at 1200×*g* for 10 min. All samples were snap-frozen in liquid nitrogen for later use or mechanically dissociated in RIPA buffer (Sigma Aldrich), called lysates (Lt), for western blot analysis.

### RNA isolation

Total RNA was isolated from frozen BMEC and SCMEC monolayers or BMVs and SCMVs using the RNeasy plus Universal Mini kit (Qiagen, Courtaboeuf, France), according to the manufacturer’s instructions. RNA concentration was determined using a Nanodrop 2000 spectrophotometer (ThermoFisher Scientific, Villebon sur Yvette, France) and RNA integrity assessed on an Agilent 2100 Bioanalyzer (Agilent Technologies, Les Ulis, France).

### Microarray assay

The transcriptome analysis of BMEC and SCMEC monolayers (stimulated or not with TNF-α) was performed on rat Whole Genome Oligo Microarrays; 40,000 genes (Agilent Technologies). Sample amplification, labeling, and hybridization were performed in line with the Agilent one-color microarray-base analysis (low input quick amp labeling) protocol (Agilent Technologies). Briefly, total RNA was reverse transcribed into complementary DNA (cDNA) using the T7 promoter primer. Synthesis of cyanine-3-labeled complementary RNA (cRNA) from cDNA was performed in a solution containing dNTP mix, T7 RNA polymerase, and cyanine 3-dCTP and then incubated at 40 °C for 2 h. Labeled cRNA was purified and fragmented before hybridization on Agilent Rat Gene Expression 4X44K Arrays (Agilent Technologies, ref: G4131F) at 65 °C for 17 h. Raw microarray signals were scanned and extracted using Agilent Feature Extraction Software (Agilent Technologies). AgiNDR package was used for quality control and normalization. Quantile methods and a background correction were applied for data normalization. Microarray data are available in the ArrayExpress database [13] under accession number E-MTAB-4696.

### Real-time quantitative PCR (RT-qPCR)

Single-strand cDNA was synthesized from 1 μg total RNA using the High Capacity RNA to cDNA Kit (Applied Biosystems, Foster City, CA, USA) according to the manufacturer’s instructions. RT-qPCR experiments were carried out with a 7500 Fast Real-Time PCR System (Applied Biosystems). All reactions were performed on 25 ng of cDNAs from BMEC and SCMEC monolayers, BMVs, and SCMVs using the TaqMan Fast Universal PCR Master Mix and different probes from the TaqMan Gene Expression Assays with the following references:

**Table:** 

Genes	ID
*Bgn*	Rn01529734
*Col3a1*	Rn01437681
*Col1a2*	Rn01526721
*Cldn9*	Rn01460292
*Spp1*	Rn01449972
*Ctgf*	Rn01537279
*Tgfb2*	Rn00579674
*Tnfrsf1b*	Rn00709830
*Mmp9*	Rn00579162
*Mmp13*	Rn01448199
*Mmp3*	Rn00591740
*Mmp12*	Rn00588640
*Mmp14*	Rn00579172
*Hoxa9*	Rn03416316
*Hoxb7*	Rn01464078
*Ccl2*	Rn00580555
*Gapdh*	Rn01775763
*Actb*	Rn00667869
*Rpl13*	Rn00821258

Samples were run in duplicates on the same 96-well plates and analyzed with the 7500 Software v2.0 (Applied Biosystems). Relative expression levels were determined according to the ΔΔCt method where the expression level of the mRNA of interest is given by 2^-ΔΔCT^ where ΔΔCT = ΔCT target mRNA – ΔCT reference mRNA (*Gapdh* for the MECs, *Actb and Rpl13* for the MVs) in the same sample.

### Western blot analysis

The lysates (Lt) from BMEC monolayers (stimulated or not with TNF-α), BMVs, and SCMVs were defrosted and centrifuged at 13,000×*g* for clarification. Protein concentrations were determined using the Lowry method (Bio-Rad, Hercules, CA, USA). After boiling, aliquots containing equal amounts of protein were loaded in Laemmli buffer and separated by 8.5 % sodium dodecyl sulfate (SDS) polyacrylamide (Bio-Rad) gel electrophoresis (PAGE) using a MiniBlot system (Bio-Rad). Proteins were transferred onto nitrocellulose membranes (Amersham Biosciences, Buckinghamshire, UK) in transfer buffer (Tris 25 mM, glycine 192 mM, ethanol 20 %). Membranes were incubated overnight in blocking buffer at 4 °C and then probed with primary antibodies diluted in blocking buffer (TBS with milk 5 % and Tween20 0.2 %). The following antibodies were used: goat anti-β-actin HRP 1/5000 (Santa Cruz, Dallas, Texas, USA), mouse anti-claudin-5 2 μg/mL (Life Technologies), rabbit anti-ZO-1 2 μg/mL (Life Technologies), rabbit anti-occludin 2 μg/mL (Life Technologies), rabbit anti-HOXA9 1.5 μg/mL (Novus biological, Littleton, CO, USA), rabbit anti-HOXB7 0.5 μg/mL (Proteintech, Rosemont, IL, USA), rabbit anti-TNFR1 0.5 μg/mL (Proteintech), and rabbit anti-TNFR2 0.5 μg/mL (Proteintech). After washing, membranes were incubated with appropriate secondary horseradish peroxidase (HRP)-conjugated IgG antibodies 1/2000 (Jackson Immunoresearch). Finally, proteins were detected using a chemiluminescence kit (Roche Diagnostics, Mannheim, Germany) revealed with the G:Box chemi xx6 system (Syngene, Cambridge, UK). Films were digitized using GeneSys software (Syngene) and optical densities of the bands were assessed using the NIH ImageJ software.

### Design of the study and data analysis

For microarray-based transcriptomic analysis, two inserts containing the BMEC or SCMEC monolayers, stimulated or not with TNF-α 5 ng/mL for 12, 24, and 48 h, were pooled for RNA isolation. The transcriptome analysis was performed twice from independent in vitro BBB and BSCB model preparations and TNF-α induction (duplicates). For the basal differential gene expression analysis, the ratio of the values in BMECs versus SCMECs or BMVs versus SCMVs was filtered based on a fold change (FC) ≥1.45. For the differential gene expression analysis in response to TNF-α, the values of the induction versus control at the same time points were filtered based on a FC ≥1.45 and <2 (genes moderately induced) or a FC ≥2 (genes highly induced). Only genes exhibiting the defined FCs for all combinations between duplicates were considered.

For BMEC or SCMEC analysis (RT-qPCR, ELISA, western blot, permeability), at least three inserts (triplicates) containing the MEC monolayers were used and all experiments were repeated at least three times from independent in vitro BBB and BSCB model preparations. All data are expressed as means ± standard deviations. The values were compared using Student’s *t* test. Results were considered statistically significant at *p* ≤ 0.05 (*), *p* ≤ 0.01 (**), or *p* ≤ 0.001 (***). BMV and SCMV analyses (RT-qPCR and western blot) were based on three independent pools of at least six rats.

### Transcript data mining

Biological interpretation of the transcriptomic data was performed using the Java/Perl software PredictSearch®, which has been previously described [14–16]. This software characterizes the pathways and functional networks in which the selected genes are involved.

## Results and discussion

### Basal differential gene expression in BMECs and SCMECs

To identify genes that presented a distinct basal expression in BMECs and SCMECs, different criteria were applied on the transcriptomic data. Only values for all controls higher than the background (according to Agilent calculations) were considered. In a first approach, ratios (BMEC values versus SCMEC) were filtered based on a fold change (FC) ≥1.45. Only genes exhibiting the defined FCs for all combinations between duplicates were considered. These criteria led to select 648 genes exhibiting a higher expression in BMECs and 444 with a higher expression in SCMECs. Further analysis indicated that a high number of genes encoding known endothelial markers [17] and adhesion/TJ molecules have a similar expression pattern in BMECs and SCMECs (Table 1). However, some of the genes related to the extracellular matrix (ECM) such as *Bgn* (biglycan), *Col3a1* (collagen type III alpha 1), *Col1a1* (collagen type I alpha 1), *Col1a2* (collagen type I alpha 2), *Slit3* (slit guidance ligand 3), *Mgp* (matrix Gla protein), *Spp1* (secreted phosphoprotein 1/osteopontin), *Ctgf* (connective tissue growth factor), and *Cldn9* (claudin-9) were among the most strongly expressed genes in BMECs (Table 1). Other highly expressed BMEC genes (data not shown) were related to either cellular messengers within the central and peripheral nervous systems: *Gal* (galanin), *Geft* (Rho guanine nucleotide exchange factor 25), *Nsg1* (neuron specific gene family member 1), *Npy* (neuropeptide Y); atherosclerosis: *Ldb2* (LIM domain binding 2), *Xdh* (xanthine dehydrogenase), *Il1rl1*/*Il33r* (interleukin 1 receptor like 1); or fatty acid metabolism: *Lpl* (lipoprotein lipase), and *Apoe* (apolipoprotein E). Among the genes encoding growth factors, only *Vegfc* (vascular endothelial growth factor C) and *Tgfb2* (transforming growth factor beta 2) exhibited a differential expression (Table 1). High basal expression of *Tgfb2* in BMECs might be correlated to the higher expression of TGF-β-target genes such as *Bgn*, *Ctgf*, and collagens [18–20].

**Table 1:** Comparative basal expression of selected endothelium-related genes in BMECs and SCMECs

Adhesion and ECM molecules			Endothelial cell markers			Growth factors		
=	>	<	=	>	<	=	>	<
*Cldn5*	*Cldn9*	*Itga2*	*Cd34*	*Cd248*	*Fabp5*	*Vegfa*	*Vegfc*	
*Cldn15*	*Ocln*		*Cd93*	*Colec12*		*Vegfb*	*Tgfb2*	
*Esam*	*Cadm1*		*Cd151*	*Klf4*				
*Cercam1*	*Bbn*		*Emcn*	*Tnfrsf1b*				
*Icam1*	*Col3a1*		*Sele*					
*Icam2*	*Col1a1*		*Dcbld2*					
*Icam4*	*Col1a2*		*Ace*					
*Pecam1*	*Mgp*		*F3*					
*Mcam*	*Slit3*		*Thbd*					
*Vcam1*	*Spp1*		*Hif1*					
*Itga1*	*Ctgf*							
*Itga4*								
*Itga5*								
*Itga6*								
*Itgae*								
*Itgav*								
*Itgb3*								
*Itgb4*								
*Itgb5*								
*Jam2*								
*Jam3*								
*Tjp1*								
*Tjp2*								
*Tjp3*								

Ratio of the values in BMECs versus SCMECs was filtered by the following FCs. FCs <1.45 and >0.69 correspond to a similar expression between BMECs and SCMECs (=). FCs ≥1.45 correspond to a higher expression in BMECs (>). FCs ≤0.69 correspond to a higher expression in SCMECs (<)

Thus, the transcriptomic analysis of SCMEC and BMEC monolayers showed differential basal expression of a significant number of genes indicative of phenotypical differences between these two CNS endothelial cell types. Indeed, distinct and characteristic gene expression profiles were found among blood vessels and MECs from different tissues [21]. For instance, TGF-β2 was reported to be higher in primary human cerebral endothelial cells (HCECs) than in human umbilical vein endothelial cells (HUVECs) at both mRNA and protein levels [22]. However, at this stage, we cannot exclude that the gene expression profile in BMECs and SCMECs resulted from the cell culture conditions or a technical bias.

To investigate whether these differences at the basal expression level exist also in vivo, RT-qPCR was performed on RNA from freshly extracted BMVs and SCMVs as the in vivo tissues closest to cultured MECs. With the exception of *Spp1*, RT-qPCR confirmed the differential expression observed in the transcriptomic analysis for *Bgn*, *Col3a1*, *Col1a2*, *Cldn9*, *Ctgf*, and *Tgfb2* (Table 2). Among these transcripts, *Cldn9* exhibited the strongest differential expression in vivo. Although the functional impact of such a difference in *Cldn9* basal expression between BMECs and SCMECs remains unclear, the level of its expression in BMECs could reflect a distinct degree of activation of signaling components. Indeed, it was shown that silencing of c-Jun NH(2)-terminal kinases (JNKs), JNK1 or JNK2, increased CLDN9 mRNA expression in epithelial cells [23]. Thus, it can be postulated that differential *Cldn9* expression might reflect different basal activities of JNKs in BMVs and SCMVs, which consequently would impact the barrier integrity through the modulation of claudins.

**Table 2:** In vivo validation of endothelium-related genes highly expressed in BMECs compared to SCMECs

Gene	Probe	MA (in vitro)	RT-qPCR (in vivo)
*Bgn*	A_43_P11812	22.9	1.5
*Col3a1*	A_44_P146518	20.9	2.3
*Col1a1*	A_44_P238421	17.2	ND
*Col1a2*	A_43_P12783	13.6	1.9
*Slit3*	A_44_P1024315	12.8	ND
*Mgp*	A_42_P588944	12.3	ND
*Cldn9*	A_44_P419898	10.0	46.2
*Spp1*	A_44_491796	4.9	0.5
*Ctgf*	A_42_P484738	4.8	2.1
*Tgfb2*	A_44_P246538	2.5	1.9

Ratio of the values in BMEC versus SCMEC monolayers (*MA* microarray) and freshly extracted BMVs versus SCMVs (RT-qPCR)

**Fig. 1:**
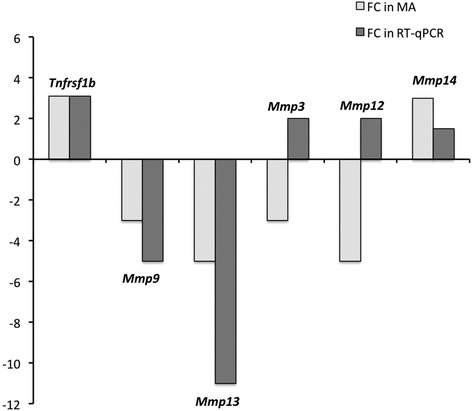
In vivo validation of differentially expressed genes between brain and spinal cord MECs. Fold changes (FCs) deduced from ratios of microarray (MA) values (BMEC versus SCMEC monolayers) were compared to FCs deduced from ratios of RT-qPCR values (freshly extracted BMVs versus SCMVs)

As shown in Fig. 1, we also observed differential expression of other genes such as *Tnfrsf1b*, *Mmp9*, *Mmp13*, and to a lesser extent of *Mmp14* in BMVs and SCMVs that followed the expression pattern deduced from the transcriptomic data in BMEC and SCMEC monolayers. In contrast, RT-qPCR for *Mmp3* and *Mmp12* in BMVs and SCMVs led to opposite values when compared to the transcriptomic analysis (Fig. 1), which might illustrate differences between cultured MEC monolayers and freshly extracted MVs.

### Basal differential expression of the HOX gene family in BMECs and SCMECs

**Fig. 2:**
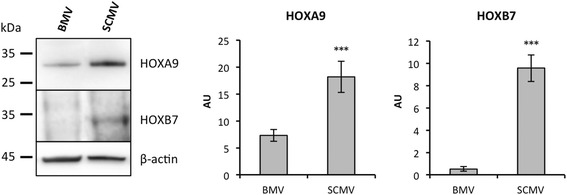
Differential protein expression of HOXA9 and HOXB7 in brain and spinal cord MECs. Western blot analysis (*left panel*) and quantification (*right panel*) of HOXA9 and HOXB7 levels were performed in whole tissue lysates from freshly extracted BMVs and SCMVs. β-actin was used to check loading of equal amounts of total protein. Quantification of the optical densities of each band was assessed using the NIH ImageJ software and is shown in arbitrary units (AU). All data are representative of at least three experiments with superimposable results. Data are presented as mean ± standard deviation (***p ≤ 0.001)

The basal differences observed between BMECs and SCMECs at the gene expression level suggested differential regulation of master program genes involved in cell differentiation. Interestingly, among the top 50 genes presenting higher expression in SCMECs than in BMECs, *Hoxa9* and *Hoxb7* were listed in the first rank and these results were confirmed using RT-qPCR (not shown). Moreover, other members of the same family exhibited a similar profile (Table 3). Similar differences were observed in vivo when *Hoxa9* and *Hoxb7* expression was assessed on mRNAs extracted from BMVs and SCMVs. Using RT-qPCR, we found higher expression levels of *Hoxa9* and *Hoxb7* mRNAs in SCMVs compared to BMVs (Table 3). Western blot performed on protein extracts generated from BMV and SCMV samples confirmed differential expression of HOXA9 and HOXB7 at the protein level (Fig. 2).

**Table 3:** Basal differential *Hox* gene expression in BMECs compared to SCMECs

Gene	Probe	MA (in vitro)	RT-qPCR (in vivo)
*Hoxa9*	A_44_P129029	0.04	0.002
*Hoxa5*	A_44_P292669	0.30	ND
*Hoxb7*	A_44_P266984	0.04	0.003
*Hoxb9*	A_44_P205783	0.60	ND
*Hoxd9*	A_44_P436218	0.20	ND

Ratio of the values in BMEC versus SCMEC monolayers (*MA* microarray) and freshly extracted BMVs versus SCMVs (RT-qPCR)

*Hox* genes encode transcriptional factors of the homeobox (HOX) protein family. Expression of these genes is involved in morphogenesis and differentiation and is spatially and temporally regulated during embryonic development. The role of HOXA9 is critical for endothelium commitment resulting from the differentiation of circulating endothelial progenitor cells into mature endothelial cells [24]. On the other hand, HOXB7 was reported to act as a key factor upregulating a variety of pro-angiogenic stimuli leading to increased matrix metalloproteinase-9 (MMP9) expression [25], which is in line with its higher expression in SCMVs (Fig. 2). Recently, a transcript analysis study revealed shared and differential patterns of *Hox* gene expression between endothelial cells from different vascular beds [26]. *Hoxd1*, *Hoxd3*, *Hoxd4*, *Hoxd8*, and *Hoxd9* were found to be expressed at a higher level in blood-derived outgrowth endothelial cells (BOECs) than in pulmonary artery endothelial cells (PAECs). It was suggested that the HOX clusters *Hoxa7-10* and *Hoxb5-7*, which were consistently expressed in BOECs, HUVECs, and human aortic endothelial cells (HUAECs), remain expressed in differentiated endothelial cells. In line with this study, our results showing a differential expression in microvessels of distinct vascular beds sustain the possibility that *Hox* genes, known as master regulators of positional identity, can define endothelial phenotypes. It is tempting to speculate that upstream epigenetic events, which are known to regulate *Hox* gene expression [27], are responsible for these different endothelial phenotypes. Indeed, *Hox* gene expression during development undergoes tight spatiotemporal regulation, partly by chromatin structure and epigenetic factors [28]. Particularly, HOXA9 expression was found to be downregulated by histone deacetylase (HDAC) inhibitors while its overexpression partially rescued the endothelial differentiation of adult progenitor cells blocked by these inhibitors [29]. Although the impact of such differences in *Hox* gene expression in SCMEC and BMEC monolayers is unclear, it can be postulated that they might influence the intrinsic capacity of MECs to respond to external stimuli, such as pro-inflammatory cytokines.

### BMEC and SCMEC inflammatory responses

**Fig. 3:**
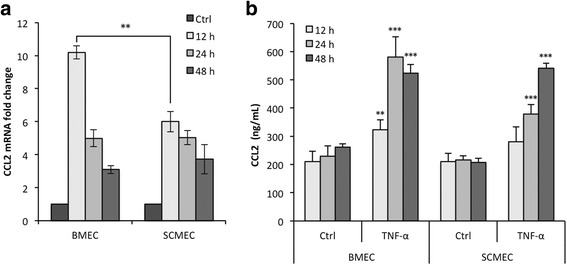
CCL2 expression and secretion upon TNF-α at 12, 24, and 48 h in BMEC and SCMEC monolayers. **a** The steady state levels of *Ccl2* mRNA relative to *Gapdh* was assessed by RT-qPCR in all tested conditions. **b** CCL2 secretion in the culture supernatants was assessed by anti-CCL2 ELISA quantification in all tested conditions. Data are presented as mean ± standard deviation (**p ≤ 0.01; ***p ≤ 0.001)

**Fig. 4:**
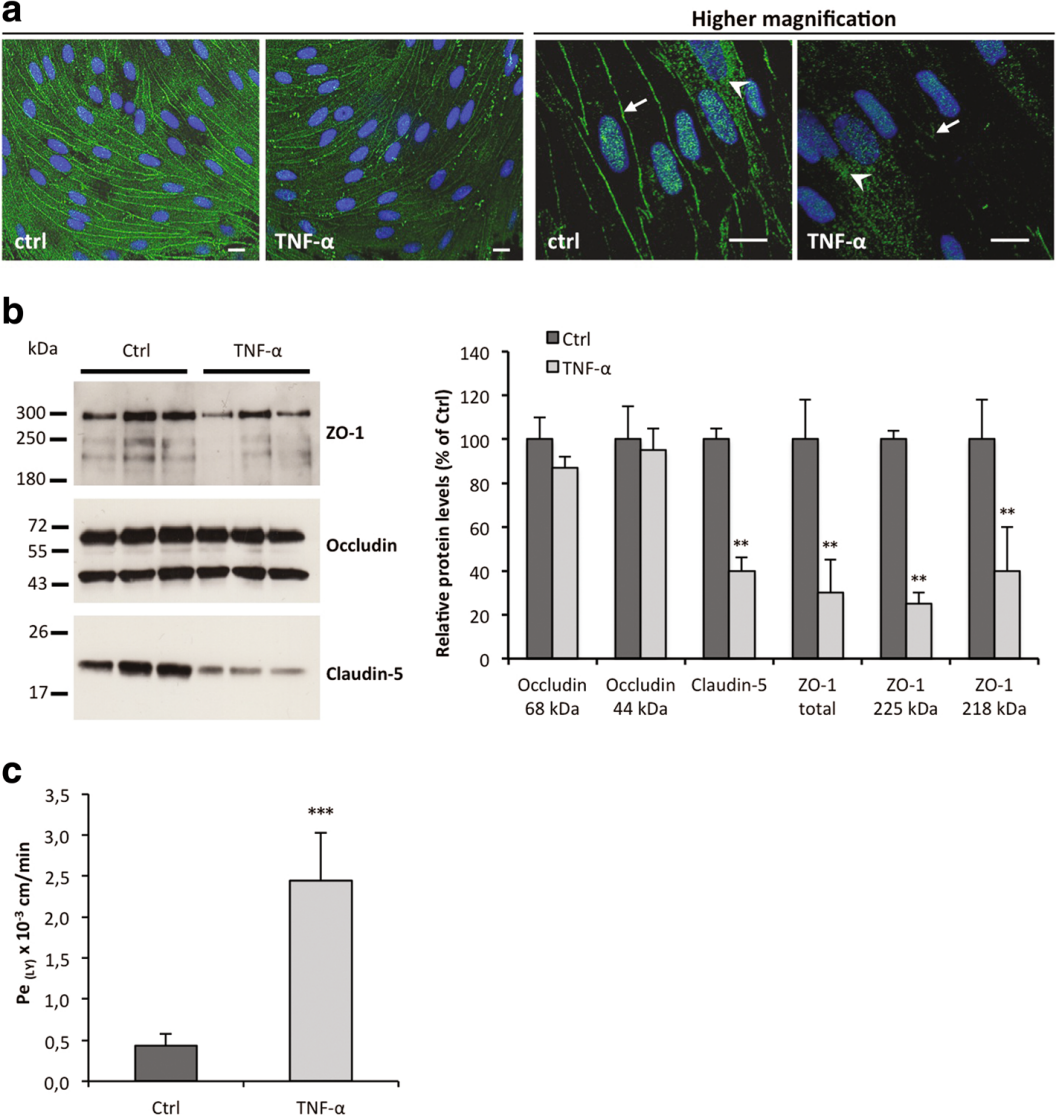
TNF-α effects on MEC monolayers integrity (24 h incubation). **a** Representative photomicrographs of BMEC monolayers fixed, permeabilized, and stained with an antibody directed against occludin (*scale bars*, 10 μm). Nuclei are stained with Hoechst. *Arrows* indicate pericellular localization of TJ protein occludin, while *arrowheads* point to cytoplasmic/vesicular of occludin. Note the decreased pericellular and increased cytoplasmic/vesicular distribution of occludin upon TNF-α treatment. **b** Western blot analysis (*left panel*) and quantification (*right panel*) of TJ proteins ZO-1, occludin, and claudin-5 levels were performed in whole tissue lysates from BMEC monolayers. Quantification of the optical densities of each band was assessed using the NIH ImageJ software. Values deduced from non-treated cultures (*Ctrl*) were considered as 100 %. **c** Barrier integrity was assessed by quantification of the Lucifer Yellow (LY) endothelial permeability, Pe_(LY)_. The loss of barrier integrity was validated for Pe_(LY)_ above 0.6.10^−3^ cm/min. All data are representative of at least three experiments with similar results and are presented as means ± standard deviation (**p ≤ 0.01; ***p ≤ 0.001)

To investigate whether rat BMEC and SCMEC monolayers respond differently to pro-inflammatory cytokines, they were either treated with TNF-α for 12, 24, and 48 h or left untreated. Validation of the TNF-α response in these cellular models was achieved by following expression and secretion of CCL2 in all tested conditions (Fig. 3). RT-qPCR showed that the steady state levels of CCL2 mRNA increased rapidly in BMECs after 12 h of TNF-α treatment and then decreased progressively from 24 to 48 h (Fig. 3a). Although a similar TNF-α response was observed in SCMECs, the level of induction at 12 h was much lower than in BMECs. The inflammatory response of BMECs and SCMECs to TNF-α was confirmed by measuring CCL2 protein levels in the culture supernatants (Fig. 3b). TNF-α-induced secretion of CCL2 was maximal at 24 h in BMECs and at 48 h in SCMECs. TJ proteins are essential in BBB homeostasis and among them, occludin, ZO-1, and claudins in different vascular beds show differential expression in development, pathology, and BBB demise [1]. Occludin immunostaining in TNF-α-treated BMEC and SCMEC (not shown) monolayers systematically showed decreased tight junction/pericellular distribution and cytoplasmic/vesicular-like distribution compared to non-treated cells (Fig. 4a). This altered distribution was not associated with overall changes in occludin steady state levels as shown by western blot analysis in BMEC monolayers (Fig. 4b). Higher doses of TNF-α (100 ng/mL) on epithelial Caco-2 monolayers followed by western blot analysis have been shown to decrease levels of phosphorylated occludin (85 kDa), but had no effect on the non-phosphorylated form (65 kDa) [30]. Our results suggest that the MEC monolayers may express mainly the non-phosphorylated form of occludin, with no effect of TNF-α on steady state levels. In contrast, western blot analysis of claudin-5 and ZO-1 (Fig. 4b) indicated decreased steady state levels of these TJ proteins. Together, changes in occludin distribution and decreased claudin-5 and ZO-1 strongly suggested disruption of MECs monolayer integrity by TNF-α. This was confirmed by the increased Lucifer Yellow (LY) paracellular leakage in the abluminal compartment as shown for the BMEC monolayer, (Fig. 4c), in agreement with previous studies [12, 31, 32].

### Transcriptomic analysis of BMECs and SCMECs in response to TNF-α

Transcriptomic analysis performed on rat MEC monolayers confirmed at the transcript level the decreased expression of *Cldn5* (claudin-5) upon TNF-α treatment at all time points in both BMECs and in SCMECs (Table 4). Such a transcriptional repression of *Cldn5* was reported to be triggered via nuclear factor kappa B (NFkB) signaling activity in retinal endothelial cells [33]. Like for *Cldn5*, expression of *Cldn9* was strongly repressed by TNF-α in both BMECs and SCMECs at nearly all kinetic time points (data not shown). This downregulation of genes involved in TJ formation is likely associated with the TNF-α-induced opening of the BBB.

**Table 4:** TNF-α modulated expression of genes encoding TJ proteins in BMECs and SCMECs

		Basal	TNF-α					
		BMEC vs SCMEC	BMEC			SCMEC		
Gene	Probe		12 h	24 h	48 h	12 h	24 h	48 h
*Ocln (a)*	A_43_P12552	**1.7**	0.7	***0.5***	***0.5***	***0.5***	***0.6***	0.9
*Ocln (b)*	A_44_P1007729	**1.7**	0.8	***0.5***	0.7	***0.5***	0.7	1.0
*Cldn5*	A_43_P15791	0.8	***0.5***	***0.5***	***0.4***	***0.6***	***0.4***	***0.4***

Ratio of the values in BMECs versus SCMECs and in TNF-α treated cultures after 12, 24, and 48 h versus non-treated cultures were filtered for FC ≥1.45 (in bold) or FC ≤0.69 (in bold and in italic)

**Fig. 5:**
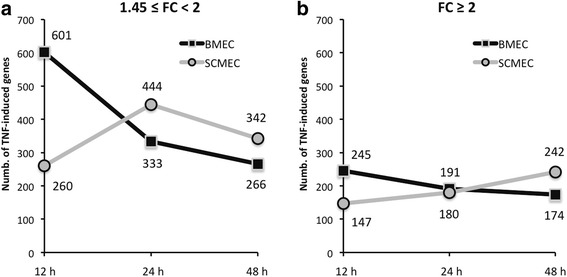
Kinetics of global transcriptomic effects of TNF-α in BMEC and SCMEC monolayers. Genes moderately induced and highly induced upon TNF-α treatment at either 12, 24, or 48 h were selected according to their FCs (**a** 1.45 ≤ FC < 2 and **b** FC ≥ 2, respectively)

Analysis of the overall transcriptomic data showed that TNF-α induction was consistently more efficient in BMECs and appeared earlier than in SCMECs (Fig. 5). Indeed, in BMECs, the number of genes whose expression was moderately induced by TNF-α (FC ≥1.45 and <2) reached a peak at 12 h, then decreased in the course of time (Fig. 5a). Within the same FC range, the maximal number of genes more strongly induced in SCMECs was higher at 24 h, then decreased at 48 h but remained slightly higher than that in BMECs (Fig. 5a). For a FC ≥2, the number of induced genes increased from 12 to 24 h in SCMECs while it decreased in BMECs (Fig. 5b). This differential induction might reflect either the presence of cell-type specific TNF-α related signaling factors or distinct basal activities of these factors in BMECs and SCMECs.

To confirm the differential TNF-α response in BMECs and SCMECs, we investigated the expression levels of several known TNF-α targeted genes such as those encoding chemokines, including CCL2 and adhesion molecules (ICAM1, VCAM1). Most of these genes exhibited a stronger induction upon TNF-α treatment in BMECs compared to SCMECs at 12 h while, except for *Ccl5*, the opposite was true at 48 h in SCMECs (Table 5). Basal expression of these genes was either similar in the two cell types or higher in SCMECs (Table 5).

**Table 5:** TNF-α modulated expression of known TNF-α targeted genes (chemokines and adhesion molecules) in BMECs and SCMECs

		Basal	TNF-α					
		BMEC vs SCMEC	BMEC			SCMEC		
Gene	Probe		12 h	24 h	48 h	12 h	24 h	48 h
*Ccl2 (a)*	A_42_P695401	***0.6***	**7.4**	**4.8**	**2.2**	**3.5**	**3.5**	**3.1**
*Ccl2 (b)*	A_42_P695407	***0.6***	**5.7**	**2.1**	**2.1**	**4.7**	**3.8**	**3.5**
*Ccl7 (a)*	A_44_P1022002	***0.3***	**11.0**	**6.7**	**3.0**	**5.4**	**4.4**	**3.9**
*Ccl7 (b)*	A_44_P391296	***0.3***	**14.0**	**6.2**	**2.8**	**5.5**	**5.0**	**4.5**
*Ccl5*	A_44_P304323	***0.2***	**7.8**	**11.0**	**16.7**	**6.5**	**7.7**	**8.0**
*Cxcl1*	A_42_P473398	0.8	**2.5**	**2.2**	1.2	**2.0**	**1.8**	**1.8**
*Cxcl2*	A_44_P5151976	0.7	**4.4**	**3.3**	1.4	**2.4**	**2.6**	**2.5**
*Cxcl3*	A_44_P363116	***0.5***	**9.8**	**7.5**	**2.7**	**6.1**	**5.0**	**3.9**
*Cxcl6*	A_44_P270366	***0.5***	**7.6**	**4.4**	**4.3**	**2.2**	**5.5**	**6.6**
*Cxcl10*	A_44_P1039128	0.7	**2.2**	1.1	1.4	**1.5**	**2.6**	**2.1**
*Cxcl11*	A_44_P175495	***0.4***	**8.6**	**7.8**	**5.6**	**5.9**	**8.9**	**6.4**
*Cxcl12 (a)*	A_44_P337351	0.9	**3.1**	**2.7**	0.8	**3.2**	**2.7**	**2.0**
*Cxcl12 (b)*	A_44_P1034439	1.1	**3.5**	1.3	0.9	**2.5**	**2.4**	**2.1**
*Icam1*	A_43_P15253	0.8	**3.0**	**3.4**	0.8	**2.0**	**3.8**	**2.3**
*Vcam1*	A_42_P499158	1.0	**7.7**	**2.0**	1.4	**3.0**	**2.4**	**2.3**

Ratio of the values in BMECs versus SCMECs and in TNF-α treated cultures after 12, 24, and 48 h versus non-treated cultures were filtered for FC ≥1.45 (in bold) or FC ≤0.69 (in bold and in italic)

Remarkably, among the top ten genes with the highest induction following TNF-α treatment at 12 h, in either BMECs (Table 6(A)) or SCMECs (Table 6(B)), several genes including *Mmp3*, *Mmp9*, *Mmp10*, *Mmp12*, and *Mmp13* that belong to the MMPs family were identified. MMPs are important in development and in numerous physiopathological processes including neuroinflammation. In the CNS, they cleave, activate, or release cytokines, growth factors, receptors, and death-inducing ligands and receptors through sheddase activity, and degrade components of the basal lamina leading to disruption of the BBB [34–36]. Most of the top ten genes exhibited a stronger TNF-α response in BMECs and, with the exception of *Ass1* (argininosuccinate synthase), *Ptgs2* (prostaglandin-endoperoxide synthase 2), and *Ptges* (prostaglandin E synthase), a higher basal expression in SCMECs (Table 6).

**Table 6:** TNF-α modulated expression of the top ten upregulated genes in BMECs (A) and SCMECs (B)

A		Basal	TNF-α					
		BMEC vs SCMEC	BMEC			SCMEC		
Gene	Probe		12 h	24 h	48 h	12 h	24 h	48 h
*Mmp3*	A_44_P318318	***0.4***	**34.9**	**9.1**	**3.1**	**7.1**	**7.0**	**5.1**
*Mmp12*	A_44_P555271	***0.2***	**33.6**	**25.6**	**7.6**	**6.6**	**6.9**	**6.5**
*Mmp13*	A_42_P606126	***0.2***	**31.4**	**8.8**	**2.4**	**5.7**	**2.9**	**3.1**
*Mmp10*	A_44_P404861	***0.2***	**16.5**	**8.8**	**10.0**	**6.9**	**8.3**	**9.7**
*Cd69*	A_43_P16166	***0.5***	**14.7**	**11.6**	**3.2**	**8.4**	**6.7**	**4.8**
*Ubd*	A_42_P602724	***0.5***	**14.6**	**10.1**	**8.7**	**4.2**	**7.4**	**5.0**
*Ccl7*	A_44_P391296	***0.3***	**13.6**	**6.2**	**2.8**	**5.5**	**5.0**	**4.5**
*Ass1*	A_44_P391296	**1.6**	**13.3**	**14.5**	**5.2**	**7.9**	**9.2**	**7.4**
*Ptgs2*	A_44_P472989	1.0	**12.5**	**10.7**	**2.9**	**5.2**	**6.5**	**3.6**
*Mmp9*	A_42_P606126	***0.3***	**12.1**	**22.2**	**22.6**	**6.4**	**5.2**	**7.5**
B		Basal	TNF-α					
		BMEC vs SCMEC	SCMEC			BMEC		
Gene	Probe		12 h	24 h	48 h	12 h	24 h	48 h
*Mmp9*	A_44_P501112	***0.5***	**10.2**	**7.9**	**9.9**	**11.7**	**20.6**	**17.6**
*Cd69*	A_43_P16166	***0.5***	**8.4**	**6.7**	**4.8**	**14.7**	**11.6**	**3.2**
*Ass1*	A_44_P391296	**1.6**	**7.9**	**9.2**	**7.4**	**13.3**	**14.5**	**5.2**
*Ptges*	A_43_P12079	1.1	**7.3**	**5.9**	**4.9**	**7.2**	**6.1**	**4.3**
*Mmp3*	A_44_P318318	***0.4***	**7.1**	**7.0**	**5.1**	**34.9**	**9.1**	**3.1**
*Mmp10*	A_44_P404861	***0.2***	**6.9**	**8.3**	**9.7**	**16.5**	**8.8**	**10.0**
*Mmp12*	A_44_P555271	***0.2***	**6.6**	**6.9**	**6.5**	**33.6**	**25.6**	**7.6**
*Ccl5*	A_44_P304323	***0.2***	**6.5**	**7.7**	**8.0**	**7.8**	**11.0**	**16.7**
*Cxcl3*	A_44_P363116	***0.5***	**6.1**	**5.0**	**3.9**	**9.8**	**7.5**	**2.7**
*Cxcl11*	A_44_P175495	***0.4***	**5.9**	**8.9**	**6.4**	**8.6**	**7.8**	**5.6**

Ratio of the values in BMECs versus SCMECs and in TNF-α treated cultures after 12, 24, and 48 h versus non-treated cultures were filtered for FC ≥1.45 (in bold) or FC ≤0.69 (in bold and in italic)

Overall, our results indicate that TNF-α induced the expression of similar sets of genes in BMECs and SCMECs with, however, distinct efficiency and/or kinetics. Such a finding in primary cell cultures is in line with the generalized notion that responses of human MECs (HMECs) and macrovascular human umbilical vein ECs (HUVECs) to inflammatory molecules are basically comparable. Nevertheless, a considerable number of genes could also be regulated in a distinct manner in different EC types [37, 38] depending on the time points. It is noteworthy to mention that most of these reports analyzing differential TNF-α induced gene expression in ECs were performed for an incubation period not exceeding 12 h. As mentioned above, one possibility is that basal expression or activity levels of signal transducers might impact the level or the delay of the TNF-α response in BMECs and SCMECs.

### TNF-α-induced expression of *Tnfrsf1b* is restricted to SCMECs

**Fig. 6:**
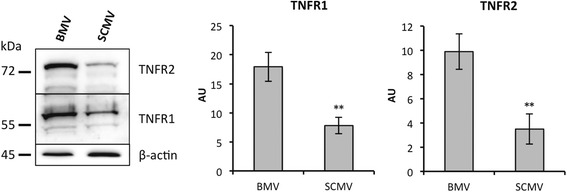
Differential protein expression of TNFR1 and TNFR2 in brain and spinal cord MECs. Western blot analysis (*left panel*) and quantification (*right panel*) of TNFR1 and TNFR2 levels were performed in whole tissue lysates from freshly extracted BMVs and SCMVs. β-actin was used to check loading of equal amounts of total protein. Quantification of the optical densities of each band was assessed using the NIH ImageJ software and is shown in arbitrary units (AU). All data are representative of at least three experiments with superimposable results. Data are presented as mean ± standard deviation (**p ≤ 0.01)

To investigate whether the delay of response in BMECs and SCMECs was correlated with differences in TNF signaling, we assessed whether genes encoding known factors involved in this pathway were modulated. Interestingly, while a slight difference was observed between BMECs and SCMECs for *Tnfrsf1a* encoding TNFR1/p55, expression of *Tnfrsf1b* encoding TNFR2/p75 was higher in BMECs compared to SCMECs (Table 7). Western blots performed on protein extracts generated from freshly extracted BMV and SCMV samples confirmed higher TNFR1 and TNFR2 protein levels (2.3- and 2.8-fold, respectively) in BMVs (Fig. 6). However, no difference was observed for genes encoding other TNF-related receptor members or products involved in the TNF signaling pathway such as *Birc2* (protein: c-IAP1), *Birc3* (protein: c-IAP2), *Tradd*, *Traff-2/3*, *Rela*, *Nfkb-1/2*, *Nfkbi-a/b*, *Ikbkb*, *Jun*, *Junb*, and *Jund* (Additional file 1: Table S1). Among all genes encoding TNF-α receptors, *Tnfrsf11b* was significantly induced by TNF-α at all time points in BMECs and at 24 and 48 h in SCMECs, while *Tnfrsf1b* was induced at 24 and 48 h in SCMECs but not in BMECs (Table 7).

**Table 7:** TNF-α modulated expression of TNF-α receptor genes in BMECs and SCMECs

		Basal	TNF-α					
		BMEC vs SCMEC	BMEC			SCMEC		
Gene	Probe		12 h	24 h	48 h	12 h	24 h	48 h
*Tnfrsf1a*	A_43_P15259	**1.5**	***0.6***	***0.6***	0.9	0.8	0.9	0.7
*Tnfrsf1b*	A_43_P533794	**3.1**	1.3	***0.6***	1.1	1.4	**2.8**	**2.3**
*Tnfrsf11b*	A_44_P458021	0.9	**4.4**	**2.4**	**2.3**	1.4	**2.9**	**2.9**

Ratio of the values in BMECs versus SCMECs and in TNF-α treated cultures after 12, 24, and 48 h versus non-treated cultures were filtered for FC ≥1.45 (in bold) or FC ≤0.69 (in bold and in italic)

TNFR1 and TNFR2 elicit distinct features [39]. Soluble TNF (sTNF) only activates TNFR1, while membrane-bound TNF (mTNF) activates both receptors [40, 41]. In contrast to TNFR1 expressed in nearly all cells, TNFR2, which can be recognized by both TNF-α and LTA (TNF-β) ligands, is limited to some cell types including endothelial cells [42]. TNFR1 and TNFR2 subunits form a heterocomplex leading to NFkB/MAPK and NFkB/PI3K-AKT-dependent NFkB/JNK signaling pathways, respectively, which trigger distinct impacts on apoptosis, proliferation, and survival [43–46]. On the other hand, it was postulated that the observed variability in TNF-induced CXCR3 chemokine expression in different microvascular beds might depend on the endothelial TNFR2 expression according to distinct anatomic loci [47].

Thus, one may speculate that the relative higher abundance of TNF-α receptors in BMECs compared to SCMECs could trigger a more rapid and stronger TNF-α response. In contrast to BMECs, SCMECs could require the TNF induction of at least *Tnfrsf1b* expression to elicit a full, albeit delayed, TNF-α response.

Noteworthy, only two other genes, *Tgfb2* and *Prkcb*, exhibited an expression pattern similar to *Tnfrsf1b*, which is higher in BMECs vs SCMECs (2.5- and 2.3-fold, respectively) and induced 2.5-fold by TNF-α at 24 and 48 h in SCMECs (not shown). It is likely that the higher basal expression of *Prkcb* in BMECs sustained the stronger and earlier TNF-α response in these cells since this gene encodes protein kinase Cβ, which plays a major role in TNF-α-induced human vascular endothelial cell apoptosis [48].

## Conclusions

The main finding of this study supports the idea that the *Hox* gene expression pattern termed in a recent report the “HOX code” [26] can define distinct endothelial phenotypes. Indeed, our data demonstrate that at least HOXA9 and HOXB7 were more abundant at the mRNA and protein levels in MECs and freshly extracted MVs from the spinal cord than those from the brain. In addition to its critical role for endothelial commitment during progenitor cell maturation [24], HOXA9 might be involved in maintaining a specific differentiation status in mature ECs through its control of the basal expression of its gene targets. In turn, HOXB7 was shown to act as a key factor to upregulate a variety of pro-angiogenic stimuli [25]. Although the impact of distinct levels of these factors in BMVs and SCMVs remains to be elucidated, it is tempting to speculate that they may control the EC response to external stimuli such as the TNF response. For instance, the involvement of HOXA9 in maintaining ECs in a “basal” state along with its inhibitory effect on NFkB-dependent transcriptional activation of endothelium has been reported [49]. Thus, while low HOXA9 abundance in BMECs might account for a sustained TNF response, its higher level in SCMECs would impair this response at an early stage, explaining the distinct efficiency and kinetics of the TNF response observed in the two cell types. At later stages, the TNF induction of *Tnfrsf1b*, specifically in SCMECs, may allow a full TNF response.

Overall our work highlights that basal gene expression is differentially regulated within ECs depending on distinct vascular beds and may account for different responses to inflammatory mediators. It can be expected that the identification of these mechanisms and the resulting functions will allow improvement of models for vascular development and plasticity as well as manipulation of EC phenotypes for therapeutic applications.

## Additional file

Additional file 1: Table S1. TNF-α modulated expression of genes involved in the TNF-α signaling pathway in BMECs and SCMECs. (DOC 53 kb)

## Abbreviations

B: Brain
FC: Fold change
MEC: Microvascular endothelial cell
MV: Microvessel
SC: Spinal cord
TJ: Tight junction
TNF: Tumor necrosis factor
