# Supplementary material for: Gene expression comparison reveals distinct basal expression of HOX members and differential TNF-induced response between brain- and spinal cord-derived microvascular endothelial cells
Source: J Neuroinflammation. 2016 Nov 10;13:290. doi: 10.1186/s12974-016-0749-6 (PMC5105278; doi:10.1186/s12974-016-0749-6)
Supplement: Additional file 1: Table S1. — TNF-α modulated expression of genes involved in the TNF-α signaling pathway in BMECs and SCMECs. (DOC 53 kb) [file 12974_2016_749_MOESM1_ESM.doc]

Additional file 1:

**Table S1** TNF-α modulated expression of genes involved in the TNF-α signaling pathway in BMECs and SCMECs

|  |  | **Basal** |  | **TNF-α** | | | | | | |
| --- | --- | --- | --- | --- | --- | --- | --- | --- | --- | --- |
|  |  | **BMEC**  **Vs**  **SCMEC** |  | **BMEC** | | |  | **SCMEC** | | |
| *Gene* | Probe |  |  | 12h | 24h | 48h |  | 12h | 24h | 48h |
| *Birc2* | A_44_P330542 | 1.1 |  | 1.2 | 1.3 | 0.7 |  | 0.9 | 1.3 | 1.3 |
| *Birc3* | A_43_P12384 | 0.9 |  | **3.2** | **2.9** | **2.1** |  | **2.5** | **2.4** | **1.9** |
| *Tradd (a)* | A_44_P527610 | 1.0 |  | 0.9 | 1.0 | 1.1 |  | 1.1 | 1.1 | 1.0 |
| *Tradd (b)* | A_42_P509843 | 1.1 |  | 1.0 | 0.9 | 1.0 |  | 1.1 | 1.2 | 1.4 |
| *Traff2* | A_44_P241277 | 0.9 |  | 1.2 | 1.2 | 1.1 |  | 1.3 | 0.9 | 0.9 |
| *Traff3* | A_44_P335636 | 1.0 |  | **1.9** | **1.8** | 0.8 |  | 1.2 | 1.3 | 1.4 |
| *Rela (a)* | A_44_P557228 | 1.0 |  | 1.1 | 1.2 | 1.0 |  | **1.6** | 1.2 | 1.1 |
| *Rela (b)* | A_44_P184726 | 1.1 |  | 1.2 | 1.1 | 0.9 |  | **1.6** | 1.2 | 1.1 |
| *Nfkb1* | A_43_P15232 | 1.1 |  | **2.6** | 1.2 | 1.3 |  | **1.6** | **1.7** | **1.8** |
| *Nfkb2 (a)* | A_42_P658264 | 0.8 |  | **1.5** | **2.2** | **1.6** |  | **2.1** | 1.4 | 1.0 |
| *Nfkb2 (b)* | A_43_P18492 | 0.7 |  | **2.4** | **2.1** | 1.2 |  | **2.2** | 1.4 | 1.1 |
| *Nfkbia* | A_44_P261450 | 1.3 |  | 1.2 | 1.1 | 1.1 |  | 1.4 | 1.4 | 1.4 |
| *Nfkbib* | A_44_P186860 | 1.1 |  | 1.2 | 1.2 | 1.1 |  | **1.5** | **1.5** | 1.1 |
| *Ikbkb* | A_43_P12782 | 1.4 |  | 1.1 | 1.2 | 1.2 |  | **1.5** | 1.4 | 1.2 |
| *Jun* | A_42_P741027 | **1.5** |  | 1.2 | ***0.5*** | 0.9 |  | 1.0 | 1.2 | 1.3 |
| *Junb* | A_43_P12125 | 1.1 |  | 0.9 | 1.4 | 1.1 |  | **2.4** | 1.3 | 1.0 |
| *Jund* | A_43_P15517 | 1.0 |  | 0.9 | 1.3 | 0.9 |  | 1.3 | 0.8 | 1.3 |

Ratio of the values in BMECs versus SCMECs and in TNF-α treated cultures after 12, 24 and 48 h versus non-treated cultures were filtered for FC ≥ 1.45 (in bold) or FC ≤ 0.69 (in bold and in italic).
